# Epithelial Cells as Active Player In Fibrosis: Findings from an *In Vitro* Model

**DOI:** 10.1371/journal.pone.0056575

**Published:** 2013-02-14

**Authors:** Solange Moll, Martin Ebeling, Franziska Weibel, Annarita Farina, Andrea Araujo Del Rosario, Jean Christophe Hoflack, Silvia Pomposiello, Marco Prunotto

**Affiliations:** 1 Institute of Clinical Pathology, University Hospital Geneva, Geneva, Switzerland; 2 Non-clinical Safety, F. Hoffmann-La Roche Ltd, Basel, Switzerland; 3 CV & Metabolic DTA Department, F. Hoffmann-La Roche Ltd, Basel, Switzerland; 4 Bioinformatics and Structural Biology Dept., Geneva University, Geneva, Switzerland; Universidade de Sao Paulo, Brazil

## Abstract

Kidney fibrosis, a scarring of the tubulo-interstitial space, is due to activation of interstitial myofibroblasts recruited locally or systemically with consecutive extracellular matrix deposition. Newly published clinical studies correlating acute kidney injury (AKI) to chronic kidney disease (CKD) challenge this pathological concept putting tubular epithelial cells into the spotlight. In this work we investigated the role of epithelial cells in fibrosis using a simple controlled *in vitro* system. An epithelial/mesenchymal 3D cell culture model composed of human proximal renal tubular cells and fibroblasts was challenged with toxic doses of Cisplatin, thus injuring epithelial cells. RT-PCR for classical fibrotic markers was performed on fibroblasts to assess their modulation toward an activated myofibroblast phenotype in presence or absence of that stimulus. Epithelial cell lesion triggered a phenotypical modulation of fibroblasts toward activated myofibroblasts as assessed by main fibrotic marker analysis. Uninjured 3D cell culture as well as fibroblasts alone treated with toxic stimulus in the absence of epithelial cells were used as control. Our results, with the caveats due to the limited, but highly controllable and reproducible *in vitro* approach, suggest that epithelial cells can control and regulate fibroblast phenotype. Therefore they emerge as relevant target cells for the development of new preventive anti-fibrotic therapeutic approaches.

## Introduction

Fibrotic diseases are characterized by the development of excess connective tissue leading to diminished organ function and eventually death. They can affect various organs, including the kidney lung, liver, heart, bone marrow, as well as skin. Millions of people are afflicted with these diseases, and, for most of them, there are few, if any, treatment options. Recognized cellular mediators of fibrosis, identified in the early 70's [1], are activated fibroblasts known as *myofibroblasts* [2]. Myofibroblasts secrete proteins of the extracellular matrix (ECM) that will progressively substitute the original functional tissue. In the kidney, fibrosis is a key player of disease progression driving nephrons loss. Moreover, the increased workload for remaining functional nephrons leads to tubular cell injury and interstitial inflammation which, by itself, is a trigger for fibrosis, creating a vicious cycle leading to progressive organ loss. Thus, progressive renal fibrosis has been clearly shown to correlate with decreasing kidney function [3].

Interestingly, recent experimental studies and clinical observations on kidney diseases [4] suggested that the induction of fibrosis might occur as a result of an altered crosstalk between tubular epithelial cells (TECs) and interstitial fibroblasts. Thus, repeated episodes of acute kidney injury (AKI) have been shown to induce fibrosis in animal models [5], [6] and AKI severity has been shown to predict progression to chronic kidney disease in humans [4]. Based on these scientific observations as well as on the histo-pathological evidence that episodes of AKI are mostly characterised by an altered TEC morphology, a causal role of epithelial cells in the induction of fibroblast activation might be hypothesized. Moreover, this hypothesis is corroborated by the common pathological observation that active interstitial fibrosis predominantly exhibits a peritubular rather than a perivascular distribution [7], [8].

Besides the mere scientific physio-pathological interest, a new pharmacological approach might emerge from this hypothesis as an alternative to the blockade of myofibroblast activation, which, at the moment, is the main pharmaceutical objective for drug hunters. Targeting fibroblast activation mediators, such as TGF-β1, has, in fact, been shown to be poorly effective and of considerable low translation between animal models [9]–[11] and humans [12].

In order to assess the role of epithelial cells in fibrosis development and, at the same time, to elaborate a simple *in vitro* system for new targets identification and drug testing, we attempted to mimic an *in vitro* model of the renal tubulo-interstitial microenvironment and acute tubular injury using a simple epithelial/mesenchymal 3D co-culture system and Cisplatin treatment. Results from our study suggest a relevant role of epithelial cells in triggering myofibroblast activation.

## Materials and Methods

### Cisplatin-treated HKC-8 cells: Preliminary experimental conditions

To mimic an acute injury, human proximal tubular epithelial cells (TECs) (HKC-8, ATCC, USA) were treated with Cisplatin, a well-known nephrotoxic drug causing DNA crosslinking and TEC apoptosis. A toxic pharmacological injury was preferred to an hypoxic insult. In order to ensure high reproducibility across all experimental groups. Experimental conditions were first determined: HKC-8 were changed to 0.5% FCS medium in the presence (or absence as a control) of several different Cisplatin concentrations. 20 µM and 40 µM Cisplatin concentrations were finally selected for further experiments as a low subtoxic dose and high toxic dose, respectively. HKC8 cells were thus incubated with these two different Cisplatin concentrations for different times (1, 2 or 4 hours). The medium was then discarded and changed to standard 2.5% FCS medium, and cells allowed recovering for 24 h, 48 h or 72 h. Cells were then sampled for assessing viability/apoptosis (see Supplemental Methods S1) . On the basis of these preliminary results, 20 µM and 40 µM Cisplatin concentrations for 4 h were selected for further analyses on *3D co-culture system.*

### Construction of the 3D co-culture system

Epithelial/mesenchymal co-culture system was composed of human proximal TECs (HKC-8, ATCC, USA) and human dermal fibroblasts (WS-1, ATCC, USA). Dermal fibroblasts were preferred to renal fibroblasts in order to mimic the *in vitro* renal tubule-interstitial microenvironment because of their low expression of fibrotic markers in basal culture conditions.. Construction of the 3D-co-culture system was performed as follows: WS-1 cells (2×10^5^ cells/gel) were mixed with rat collagen type I (see Supplemental Methods S1). Collagen gels were allowed to form at 37°C in 6-well plates, taking care to avoid the attachment of the gels to the well border. This was in order avoid tension generation and increased basal level of fibrotic markers [13]. HKC-8 cells (3×10^5^ cells) were then layered on top of the solidified collagen gels containing the WS-1 cells and 3D co-culture system cells were allowed to grow overnight. To exclude epithelial cell contamination of the gel, HKC8 cells were preloaded with Hoechst dye and microscopically evaluated within the collagen gel.

### 3D co-culture system after Cisplastin-treatment: biological analyses of HKC-8 and in gel-embedded WS-1 cells

**Figure 1:**
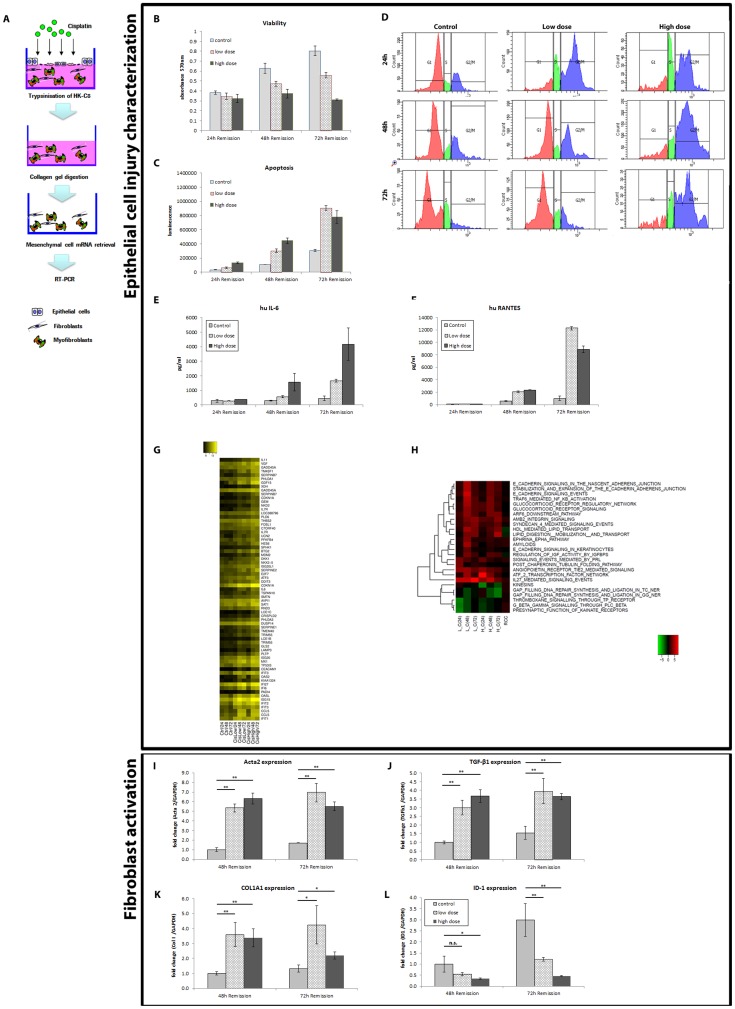
Epithelial cell injury characterization (upper panel) and fibroblast activation (lower panel) in an *in vitro* reconstructed microenvironment. (**A**) Scheme of the reconstructed microenvironment and workflow analysis of the cisplatin-injured proximal tubular epithelial cells HKC-8 cells and of the WS-1 dermal fibroblasts. (**B**) Cell viability and (**C**) apoptosis analysis. Cisplatin-treated proximal tubular epithelial cells HKC-8 cells showed decreased cell viability and increased apoptosis. (**D**). Cell cycle analysis showed that HKC-8 cells treated with cisplatin high dose (40 µM) were blocked in G2/M phase at 24, 48 and 72 h, whereas cells treated with the low dose (20 µM) reverted at 72 h to a condition similar to control. Cytokine release analysis with (**E**) IL-6 and (**F**) RANTES levels. Cisplatin-treated HKC-8 cells produced increased amounts of IL-6 and RANTES. (**G**) Gene-level analysis results for selected genes showing a stronger response to Ciplatin high dose (CisHigh) than to Ciplatin low dose (CisLow). Expression levels on a logarithmic scale are shown as a heat map: no detectable expression is indicated by black color, increasing expression levels are indicated by brighter shades of yellow. Note that several genes show up twice in the figure because they are represented by multiple probes on the Illumina chip. While the measured values do not necessarily agree, the overall trend of up-regulation is the same. (**H**) Gene-level analysis was complemented by a network-level approach using Gene Set Enrichment Analysis against the Pathway Commons collection of gene regulatory networks (www.pathwaycommons.org). Cisplatin treated cells (L: low, H: high) were compared to controls (C), and renal clear cell carcinoma (RCC) cells were compared to “normal adjacent” tissue (GEO accession number GSE781; as this data set is based on a different expression array technology, we did not compare expression levels of individual genes for this analysis). The heat map shows FDR-corrected q values on a logarithmic scale for up-regulated (red shades) and down-regulated networks (green shades), black indicating no change. An FDR-corrected q value of 0.01 corresponds to an absolute score of 4.6 on this scale. Please, note that the RCC dataset (last column) does not imply any involvement of the networks shown here. (**I–L**) RT-PCR analysis and mRNA levels of the (**I**) *Acta2* gene (encoding alpha smooth muscle actin) (**J**) *TGF-b1*gene (encoding transforming growth factor beta 1), (**K**) *COL1A1* gene (encoding collagen-1α1) and (**L**) ID-1 gene (encoding Inhibitor of differentiation 1). Retrieved WS-1 dermal fibroblasts showed increased level for key fibrotic markers α-SMA, TGF-β1 and Collagen 1α1 and decreased level of ID-1 when epithelial cells HK-C8 cells were layered on top. Gene expression profile for the same gene in absence of HK-C8 cells can be found in Figure S2B-E. n.s. = not statistically different, * = p<0.05, ** = p<0.001.

The experimental procedure is shown in Figure 1A.

Cisplatin subtoxic (20 µM) or high toxic (40 µM) doses for 4 hours exposure was added to the 3D co-culture system after careful removing the standard 2.5% FCS medium. The Cisplatin solution was then discarded and changed to standard medium. Biological analyses were conducted on the Cisplatin-treated HKC-8 cells for each recovering time, *i.e.* 24 h, 48 h and 72 h and on the in gel-embedded WS-1 cells at 48 h and 72 h after Cisplatin exposure. This time-course was chosen as a compromise between the technical limitations of our *in vitro* system and the relevant time-points of tubular injury and renal function impairment and recovery as described in an *in vivo* AKI model [14]. HKC-8 cells and WS-1 cells embedded in collagen gels were retrieved respectively by trypsinisation and by subsequent collagenase gel digestion (see Supplemental MethodsS1).

Biological analyses on the Cisplatin-treated HKC8 cells included cell cycle analysis, cytokine release and gene expression profile (see Supplemental Methods S1). This gene-level analysis was complemented by a network-level approach using Gene Set Enrichment Analysis [15] (see Supplemental Methods S1). Moreover, the retrieved Cisplatin-injured HKC8 cells were submitted to ItraQ for proteomic analysis (see Supplemental Methods S1). Characterization of fibroblast WS-1 phenotype after Cisplatin-induced epithelial injury was performed using RT-PCR analysis (See Supplemental MethodsS1).

## Results

### 1. TEC (HKC-8 cells) analysis after Cisplatin-treatment (Figure 1-upper panel)

Cisplatin-treatment induced significant HKC-8 cell death (Figure 1B) and apoptosis (Figure 1C) in all conditions. Cell cycle analysis showed marked arrest in G2/M phase at 24 h for both low and high Cisplatin doses (Figure 1D) (for Cisplatin dose- and time-course, see Figure S1A–F). Low (subtoxic) dose cisplatin-treated cells recovered slowly to G0/G1 phase (48 h and 72 h), while high (toxic) dose Cisplatin-treated cells showed irreversible G2/M phase block. Cytokine release analysis showed a marked increase of IL-6 (Figure 1E) and RANTES (Figure 1F) in all groups. MCP-1 and IL-8 levels did not change (data not shown).

At the gene level (Figure 1G and Figure S1H), Cisplatin-treatment induced overall similar effects on epithelial HKC-8 cells with both doses, a stronger response being observed with the high toxic dose:

At the early time point (24 h), CDKN1A (p21CIP1) a gene known to stop cell-cycle progression [16] was the most strongly up-regulated gene, followed by GDF15, a member of the TGF beta superfamily reported to be elevated in patients with renal diseases [17], [18]. Other up-regulated genes included interferon-inducible genes, such as ISG15, IFIT1, IFIT2, IFIT3 (data not shown), as well as genes involved in stress response, such as GADD45A and ATF3;
The intermediate time point (48 h) was characterized by the strongest effects on overall gene expression levels. GDF15 exceeded CDKN1A as the most strongly up-regulated gene (Figure S1H). CCL5 (RANTES) and IL6 were similarly up-regulated by both doses, as was TP53I3, a P53 target gene involved in cell death. Other up-regulated genes such as SERPINB7 (Megsin), TM4SF1 (a member of the tetraspanin family), VGF (nerve growth factor inducible), and DKK1 (an inhibitor of WNT signaling) were preferentially increased at the high dose;
At the late time point (72 h), gene expression changes were smaller compared to the 48 h time point, indicating an overall trend to return to levels found in the control group at low dose, but still up-regulated at high dose, as observed for GDF15 and CDNK1A.

Furthermore, Gene Set Enrichment Analysis (Figure 1H) showed that:

At the early time point (24 h) both doses elicited a strong response in interferon and cytokine networks. The P53 network was up-regulated by both doses, but much stronger for the high dose, as was the ATF2 stress response;
At the intermediate time point (48 h), the P53 network remained strongly activated for both doses, and PI3K signaling was strongly up-regulated. Interestingly, while the overall responses were still more prominent for the high dose, some networks were more significantly up-regulated for the low dose including IL27-mediated signaling, E-cadherin signaling, the nectin adhesion pathway and the RIG-I/MDA5 response;
At the late time point (72 h), IL27-mediated signaling remained strongly up-regulated for the low dose only, and E-cadherin and nectin signaling returned to lower activation levels as judged by the expression changes of their member genes.

In regard to the ATR response to replication stress and cell cycle, the networks were suppressed by the high dose at 48 h and 72 h.

Finally, we asked whether networks and genes that were specifically affected in our epithelial cell injury experiment were relevant or not for proliferation effects in cancer. For that purpose, comparative network analysis was performed with public available data set activated in clear cell carcinoma (RCC), which is the most frequent renal carcinoma. Many regulatory networks were found up-regulated in the cancer data set (*e.g.*, P53 networks, PI3K signaling, cytokine signaling), while others were apparently not affected. Figure 1H highlights some networks affected in our experiment but not in the RCC data set. Proteomic analysis confirmed variations, at protein levels, of some genes retrieved in the microarray analysis (Table S1).

### 2. Fibroblasts (WS-1 cells) analysis after Cisplatin-treatment (Figure 1- lower panel)

RT-qPCR analysis showed that, at both Cisplatin doses (low and high doses) and at both selected follow-up times (48- and 72 h), several prototypical markers of activated myofibroblasts, *i.e.* α- smooth muscle actin (SMA) (Figure 1I), TGF-β1 (Figure 1J) and collagen type I (Figure 1K), were statistically significantly increased, whereas inhibitors of differentiation or DNA binding-1 (ID-1) (Figure 1L) was significantly downregulated. Unexpectedly, CTGF was decreased at both Cisplatin doses (data not shown).

Remarkably, Hoechst dye tracing of preloaded HKC-8 cells and subsequent imaging of the collagen gel after Cisplatin-treatment showed no sign of fluorescence within the collagen gel (data not shown), attesting that increase in fibrotic markers was uniquely due to mesenchymal cell phenotypical modulation. Moreover, in order to ensure that these modulations were mediated by Cisplatin-injured epithelial cells, and not due to a Cisplatin-direct toxic effect on fibroblasts, the same experiment was performed on collagen-gel containing only WS-1 fibroblasts in the absence of HKC-8 cells. No, or only minimal modulations of the prototypical markers of activated myofibroblasts were observed (Figure S2).

## Discussion

Our results validated a simple, controlled and reproducible *in vitro* 3-D co-culture system mimicking the tubulo-interstitial renal microenvironment that could be used for further application in anti-fibrotic drug discovery. Our analysis of epithelial cell biological modulations upon Cisplatin injury is probably one of the most comprehensive analysis of gene expression profile and proteomic analysis. Most interestingly, and arising from the technical achievement that was central in our work, this *in vitro* study strongly supports the relevance of injured epithelial cell as a trigger for resting fibroblast differentiation toward an activated myofibroblast phenotype.

Our *in vitro* findings suggest that tubular epithelial cells constitute together with interstitial fibroblasts an *epithelial/mesenchymal unit* whose alteration as a whole, in our case represented by cisplatin-induced epithelial injury, is *per se* the cause of myofibroblast differentiation and activation.

It is however important to emphasize that our experimental design has several limitations. Firstly is the adoption of human dermal fibroblasts in the *in vitro* 3-D co-culture system instead of renal fibroblasts. The authors' intention was to maximize differences in fibrotic readout in order to assess the concept of epithelial cell relevance in the fibroblast phenotype control. Our preliminary experiments showed that reliable and reproducible results were possible only using dermal fibroblasts that, as stated before, express lower levels of fibrotic markers in basal conditions. Ideally, experiments would have to be repeated with primary human renal fibroblasts or human renal fibroblast cell lines. Unfortunately, our initial experiments using primary renal fibroblasts were not conclusive, due to the great level of variability in the results (data not shown) which was incompatible with the data reproducibility required for compound profiling. In addition, the unique available renal fibroblast kidney cell line is, at the moment, not available to pharmaceutical scientists.

The aim of this study was not to recreate the exact *in vivo* pathological microenvironment and even less to recreate an *in vivo* Cisplatin-induced toxicity. As mentioned in the Material and Methods section, Cisplatin was adopted to insure a high reproducibility across all the experiments., It is also noteworthy that other cell types (*e.g*., leukocytes, M1 and M2 macrophages) have a well-recognized key role in the pro-fibrotic renal microenvironment [19]. We think that, in the future, such a 3D co-culture system might also be used to address contribution of other relevant cell types. Therefore, even if the core message of this short communication is that epithelial cell injury is a causal trigger for fibroblast modulation toward an activated myofibroblast phenotype, we do not exclude that other cell types might control also myofibroblast activation as well.

Our *in vitro* findings support the relevance of epithelial cells as a master regulator of myofibroblast activation documented by other authors in kidney [20]–[22] and in other organs such as liver [23]. Several former [24]–[26] and more recent [27], [28] experimental findings, together with the common histopathological notion that regions of active interstitial fibrosis predominantly exhibit a peritubular rather than a perivascular distribution [7], [8], suggest a causal role for tubular epithelial cells in the proliferation and activation of myofibroblasts leading to renal fibrosis. Thus, experimental work by Nath *et al.* [5] showed that acute insult induced through repetitive exposure to heme proteins, mainly impacting on TECs, was invariably accompanied by a long term renal loss of kidney function associated with chronic tubulo-interstitial damages, as measured by collagen deposition and TGF-β1 activation. Using a tetracycline-controlled transgenic mouse model, Koesters *et al.* [20] demonstrated that conditional overexpression of TGF-β_1_ confined to renal TECs induced widespread peritubular proliferation of resident fibroblasts, differentiation into myofibroblasts and subsequent proliferation, and progressive deposition of ECM. Moreover, the causal association between acute epithelial injury and fibroblast activation with consequent fibrotic outcome was documented in a number of AKI experimental models, including ischemic, toxic and obstructive models [29]. Yang *et al.* convincingly showed that injured TECs, stopped in the G2/M phase of the cell cycle, released pro-fibrotic cytokines, and that bypassing the G2/M arrest by administration of a p53 inhibitor reduced fibrosis. In our small *in vitro* system , we were able to recapitulate epithelial cell G2/M phase arrest, increase in cytokine release and TEC-induction of mesenchymal cell differentiation/activation in terms of increased established pro-fibrotic markers, *i.e.*, SMA, TGF-β1 and Collagen type I. Finally, the relevance of TEC injury as a causal trigger for fibrosis progression has been elegantly demonstrated *in vivo* by Grgic *et al.* [6]. These authors developed a mouse model of kidney injury using the Six2-Cre-LoxP technology to selectively activate expression of the simian diphtheria toxin (DT) receptor in renal epithelia derived from the metanephric mesenchyme. By adjusting the timing and dose of DT, a highly selective model of tubular injury was created. Thus, Grgic *et al.* were able to show that insults to renal epithelial cells at 1-week intervals resulted in maladaptive repair with interstitial capillary loss, fibrosis, and glomerulosclerosis, which was highly correlated with the degree of interstitial fibrosis.

We think that Cisplatin-induced TEC injury adopted in our 3-D co-culture system might be an interesting model for further studying epithelial/mesenchymal crosstalk *in vitro*. Indeed, using pathway and gene analyses, we could identify in Cisplatin-injured HKC-8 cells not only several target genes known to be expressed in injured renal tubular epithelium (P21, P53, E-Cadherin) [30]–[33] , but also new target genes, such as nerve growth factor which was recently shown to exert pro-fibrotic activity independently of TGF-β1 in an *in vivo* model of fibrotic disease[34]. Finally, we observed that GDF-15, a member of the TGF beta superfamily, recently reported to be elevated in patients with renal disease[17], [18], [35], was one of the most strongly affected genes in our *in vitro* system. Interestingly, elevated urinary excretion of GDF15 [35] is associated with proximal tubular injury, a clear determinant of tubulo-interstitial inflammation and fibrosis in diabetic nephropathy [36], [37].

In conclusion, our work has allowed the construction of a highly reproducible 3-D co-culture system for further compound profiling and, with the caveats due to the limited *in vitro* approach, suggests that, in addition to treatment of renal fibrosis by antagonism of myofibroblasts activation pathways, epithelial cells might emerge as relevant targets for the development of new and preventive therapies. Further studies, in particular morphological analysis with advanced microscopy techniques, are however needed to validate these results as well as the concept of an *epithelial-mesenchymal unit* in the scientific community, not only in the renal research but also in other fibrotic diseases such as idiopathic pulmonary fibrosis.

## Supporting Information

Figure S1 **(A–F) Time-course of cisplatin injury on proximal tubular epithelial cell HKC-8 (4 h, 2 h and 1 h).** (**A–C**) Cell viability and (**D–F**) cell apoptosis. (**G**) Gene expression changes with cisplatin low dose relative to control (x axis) in comparison with cisplatin high dose relative to control (y axis) at 48 hr. Expression changes are indicated as differences on a logarithmic scale to the basis of two (“log fold changes”). Some genes of interest are labelled. Major diagonal is given to highlight the overall stronger effects for the cisplatin high dose (y axis). (**H**) Genes annotated as belonging to the “secretome” with a general upward trend with cisplatin high dose in comparison with low dose. See legend of Figure 1A for more details.
(TIF)

Figure S2 **(A–D) RT-PCR analysis of retrieved WS-1 dermal fibroblasts collected with the same procedure as in** **Figure 1I-L** **, in absence of HK-C8 cells layered on top.** (**A**) mRNA levels of the *Acta2* gene (encoding alpha smooth muscle actin), (**B**) *TGF-b1*gene (encoding transforming growth factor beta 1), (**C**) *COL1A1* gene (encoding collagen-1α1) and (**D**) ID-1 gene (encoding Inhibitor of differentiation 1) Cisplatin treatment had only slight effects on WS-1.
(TIF)

Methods S1 **Supplemental Material.**
(DOC)

Table S1 **Proteomic analysis of Cisplatin-injured epithelial cells at different time-points.** Asterisk indicates upregulated genes present also in figure 1G, - indicates Unidentified protein, NQ indicates Identified by not quantified proteins and NS indicates Not statistically significantly differentially modulated protein. Differential expression measured within each time-point is indicated with the following color code.
(DOC)
