# Supplementary figures and images for: Epithelial Cells as Active Player In Fibrosis: Findings from an In Vitro Model
Source: PLoS One. 2013 Feb 14;8(2):e56575. doi: 10.1371/journal.pone.0056575 (PMC3572957; doi:10.1371/journal.pone.0056575)

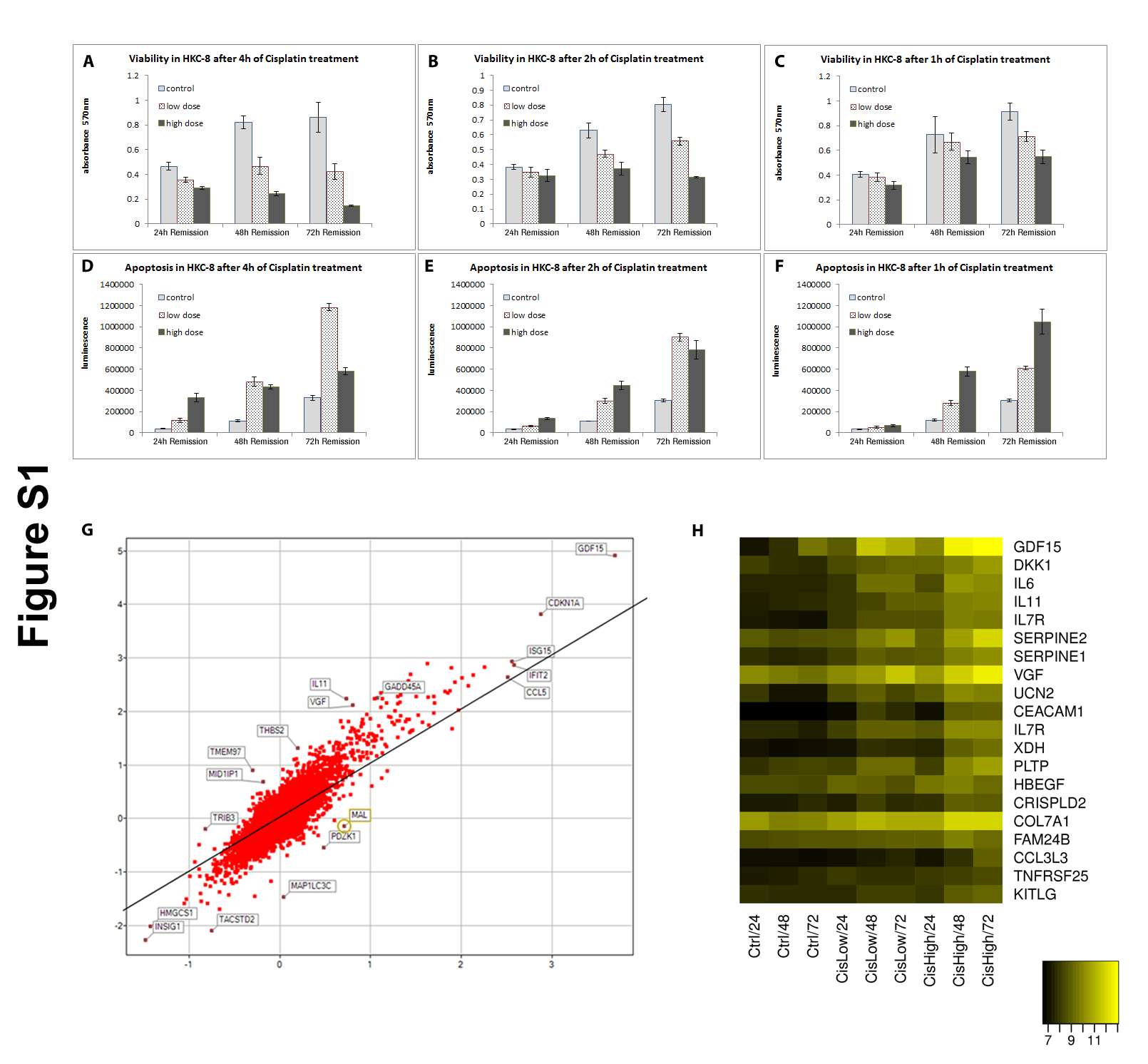

Supplement: Figure S1 — (A–F) Time-course of cisplatin injury on proximal tubular epithelial cell HKC-8 (4 h, 2 h and 1 h). (A–C) Cell viability and (D–F) cell apoptosis. (G) Gene expression changes with cisplatin low dose relative to control (x axis) in comparison with cisplatin high dose relative to control (y axis) at 48 hr. Expression changes are indicated as differences on a logarithmic scale to the basis of two (“log fold changes”). Some genes of interest are labelled. Major diagonal is given to highlight the overall stronger effects for the cisplatin high dose (y axis). (H) Genes annotated as belonging to the “secretome” with a general upward trend with cisplatin high dose in comparison with low dose. See legend of Figure 1A for more details. (TIF) [file pone.0056575.s001.tif]

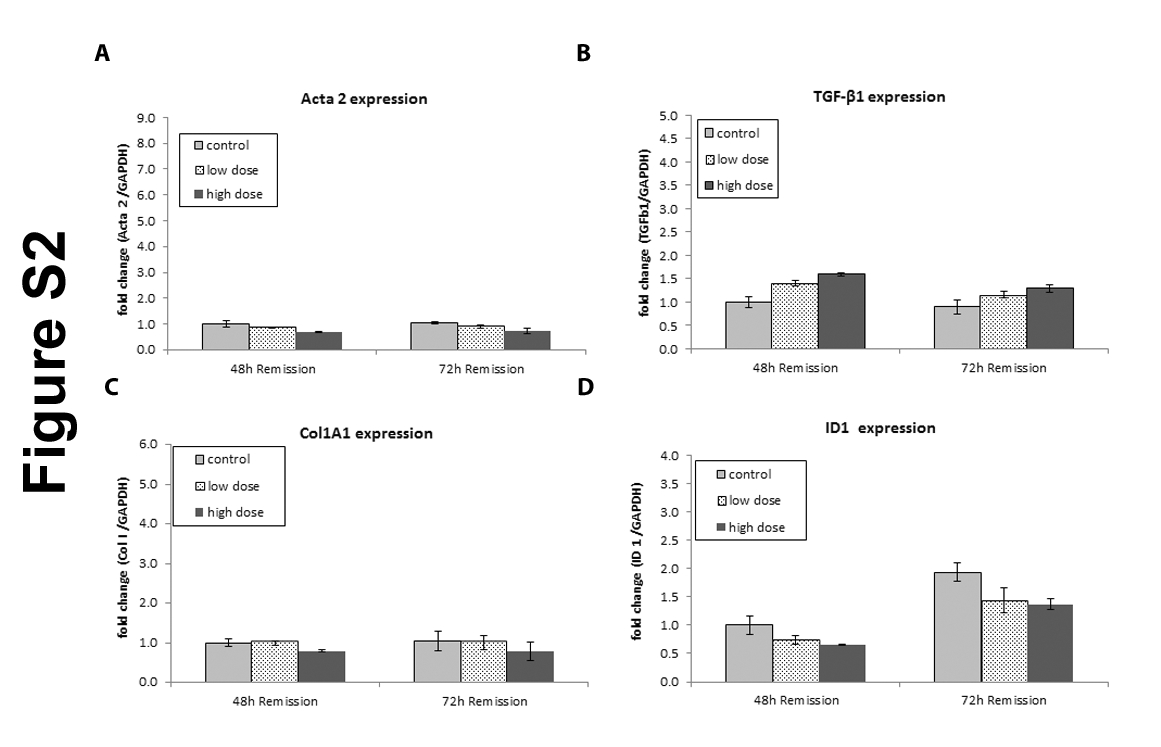

Supplement: Figure S2 — (A–D) RT-PCR analysis of retrieved WS-1 dermal fibroblasts collected with the same procedure as in Figure 1I-L , in absence of HK-C8 cells layered on top. (A) mRNA levels of the Acta2 gene (encoding alpha smooth muscle actin), (B) TGF-b1gene (encoding transforming growth factor beta 1), (C) COL1A1 gene (encoding collagen-1α1) and (D) ID-1 gene (encoding Inhibitor of differentiation 1) Cisplatin treatment had only slight effects on WS-1. (TIF) [file pone.0056575.s002.tif]
