# Supplementary material for: Epithelial Cells as Active Player In Fibrosis: Findings from an In Vitro Model
Source: PLoS One. 2013 Feb 14;8(2):e56575. doi: 10.1371/journal.pone.0056575 (PMC3572957; doi:10.1371/journal.pone.0056575)
Supplement: Methods S1 — Supplemental Material. (DOC) [file pone.0056575.s003.doc]

**Supplemental Methods**

*Cell culture*

Human proximal tubular cells HKC8 (Johns Hopkins University, Baltimore, ML, USA) were cultured in DMEM/F12 media supplemented with 2.5% FBS, 1% Insulin-Transferrin-Selenium (ITS, BD 354352), Penicillin (100 Units/ml) and Streptomycin (100µg/ml) at 37°C and 5% CO_2_.

Normal skin fibroblast WS-1 (ATCC, CRL-1502) were cultured in MEM supplemented with 10% heat-inactivated fetal bovine serum (FBS), non-essential Amino Acids (Gibco, 11140), Penicillin (100 Units/ml) and Streptomycin (100µg/ml) at 37°C and 5% CO_2_.

*Cisplatin protocol*

A Cisplatin solution (Sigma Aldrich, #P4394) was prepared freshly before use at 3.3mM in NaCl 0.9% and diluted to a 20uM and 40uM concentration in assay medium [DMEM/F12 Medium (Invitrogen, #31331) supplemented with 1%ITS (BD # 354352), 1%Pen/Strep, 0.5% FBS].

*Apoptosis and viability analysis of Cisplatin-treated HKC-8 cells*

HKC-8 cells where seeded at 10‘000 cells/well in 96-well plates. Cells were treated with Cisplatin solution for 1h, 2h or 4h and allowed to recover for 24h, 48h or 72h. Apoptosis assay (Promega Luminescence assay # G8093 Caspase 3/7 Glo) and viability assay (Promega Luminescence assay # G7571 Cell Titer Glo) were performed according to the manufacturer's instructions. Briefly, kit buffer was mixed with the lyophilisate, 100ul was added to each well, incubated for 10 minutes and Luminescence was read with EnVision reader (Perkin Elmer, Boston, USA).

*3D co-culture system preparation*

Rat tail collagen type I stock solution (GIBCO, A10483-01) was slowly diluted in cold MEM medium supplemented with NaHCO_3_ and NaOH to reach a 2mg/ml collagen solution, 1x MEM, 0.15% NaHCO_3_, 0.1N NaOH, pH 6.5-7.5, kept on ice. WS-1 cultured cells were trypsinized and re-suspended in grow medium to a concentration of 4x10^5^ cells/ml. Collagen solution and WS-1 cell suspension were mixed 1:1 to get a final solution with 1mg/ml Collagen type I and 2x10^5^ cells/ml. This collagen-cell suspension was dispensed in a pre-warmed 6-well plate (1ml per well), paying attention to avoid border contact and air bubble formation. After 5’ incubation at room temperature, plate was incubated for 4h at 37°C and 5% CO2.

In the meantime HKC-8 cultured cells were trypsinized and re-suspended in grow medium to a concentration of 1.5x10^5^ cells/ml. This cell suspension was transferred on top of the pre-formed Collagen-WS-1 gel (2ml per well) and incubated at 37°C and 5%CO_2_ overnight.

*Cisplatin treatment of the 3D co-culture system*

After careful removal of the culture medium from the 3D co-culture system, 2ml of the prepared Cisplatin solution (or medium alone as control) was added to the system which was incubated for 4h at 37°C 5%CO_2_. After incubation, supernatant was removed and the system was washed carefully twice with PBS. 2ml of culture medium was added to each well and the system was incubated for 24h, 48h and 72h.

At each follow up time, supernatants were collected for further cytokine release analysis (*see corresponding Supplemental Materials section*), and cells were harvested as follow: HKC8 cells were washed 1x with PBS. Then, 0.5 ml of 0.05% Trypsin/EDTA (Gibco # 25300) was added around collagen gels to detach the cells adherent on plastic and not in direct contact with the collagen gel. These cells were washed away with 1ml PBS and discarded. The HKC8 cells were detached from the top of the gel using trypsinisation [2ml of 0.25% Trypsin/EDTA (Gibco # 25200)]. After an incubation at 37°C for approximately 10’, the HKC8 cells were collected with 2ml culture medium and transferred to a centrifuge tube. The WS-1 cells were then collected from the gel using collagenase gel digestion: the gel was washed 1x with PBS and incubated with 2ml of collagenase solution [collagenase I (Gibco #1700-017); 100U/ml in HBSS (Gibco 14175)] until dissolution. WS-1 cells were collected from the dissolute gel with 2ml culture medium and transferred to a centrifuge tube. HKC8 and WS-1 cells were spin down, washed 1x with PBS and stored at -80°C for further analyses.

*HKC-8 cell cycle analysis (FACS) after Cisplatin-treatment*

Harvested HKC-8 cells were re-suspended in 0.5 ml PBS until a monodisperse cell suspension was achieved. Cells were then transferred into tubes containing 70% Ethanol, fixed for 2h and centrifuged for 5’ at 200 × *g*. The pellet was diluted in 5 ml PBS, allowed to equilibrate for 1’, subsequently centrifuged for 5’ at 200 × *g*, re-suspended in a PI/Triton X-100 staining solution in presence of RNase A and incubated for 30’ at room temperature. Fluorescence was measured at 617nm with excitation at 536nm using EnVision reader.

*Supernatant cytokine release analysis after Cisplatin-treatment*

At each follow up time (24h, 48h and 72h) after Cisplatin-treatment, supernatants were collected for further cytokine release. IL-6 (BioRad, 171-B5006M), IL-8 (BioRad, 171-B5008M), RANTES (BioRad, 171-B5025M) and MCP-1 (BioRad, 171-B5019M) were measured using Multiplex technology. The Multiplex assays were designed on magnetic beads according to a sandwich immunoassay format (Bio-Plex, Bio-Rad, Hercules, CA, USA). The capture antibody-coupled beads were incubated with supernatants and antigen standards samples or controls for 60’ and then incubated with biotinylated detection antibodies for 30’. After washing away the unbound biotinylated antibodies, the beads were incubated with a reporter Streptavidin-Phycoerythrin (SA-PE) conjugate for 10’. Following the removal of excess SA-PE, the beads were passed through the Bio-Plex suspension array reader (Bio-Rad, Hercules, CA, USA), which measured the fluorescence of the beads and of the bound SA-PE. All washes were performed using a Bio-plex Pro wash station (Bio-Rad, Hercules, CA, USA). Data acquisition and analysis were performed using Bio-Plex Manager 6.0 at a low PMT (Bio-Rad, Hercules, CA, USA).

*HKC-8 Affymetrix oligonucleotide DNA microarray hybridization analysis after Cisplatin-treatment*

RNA was extracted using QIAshredder and RNeasy kits (QIAGEN). 1 µg of total RNA was employed for the synthesis of biotinylated cRNA, and 1 µg of this cRNA was hybridized onto Affymetrix Human Genome U133 Plus 2.0 arrays (following Affymetrix protocol). Microarrays were normalized based on the mean of the signals of the entire microarray set. Probe sets with a normalized signal of ≥20 arbitrary units of fluorescence were considered above threshold and therefore expressed. To identify differentially expressed probe sets, pairwise comparisons between mean expression values in any treatment group and the time-matched vehicle control group were carried out for all expressed probe sets using an F-test in a one-way analysis of variance at *P*< 0.05, with Bonferroni’s correction for large-scale multiple testing. In addition, a cut-off of ≥2-fold change in magnitude *versus* the time-matched control group was applied to pairwise group comparisons. Thus, “significant” probe sets are expressed probe sets with a ≥2-fold magnitude of change in mean expression versus mean expression of the time-matched control group, and P<0.05.

*HKC-8 gene network analysis after Cisplatin-treatment*

The analysis was performed on expression data collected using the Illumina platform. For each probe on the chip, average expression levels were obtained, and the derived changes plus their statistical significance were calculated. Using the complete, ranked list of calculated changes, Gene Set Enrichment Analyses (GSEA) was run on gene sets provided by REACTOME and other sources through the Pathway Commons collection *(*[*www.pathwaycommons.org*](http://www.pathwaycommons.org/)*).* For each such set, a significance level (technically, an FDR-corrected q-value) was calculated to indicate the joint up- or down-regulation of its member genes. The collection contains around 1,000 gene sets which are partially overlapping and redundant.

An additional analysis was also run to compare expression levels of genes in a renal clear cell carcinoma (RCC) to those in normal adjacent tissue. This second data set is based on the Affymetrix technology, hence absolute levels of gene expression should not be directly compared to the first study. However, differential changes were calculated and GSEA run on the Pathway Commons collection to try and relate the two data sets to each other at the network level.

*HKC-8 ItraQ for proteomic analysis after Cisplatin-treatment*

Proteins were extracted by sonication in PBS, digested with trypsin and used for comparative proteomic analysis as previously described. Briefly, tryptic peptides from each sample were tagged with one of the isobaric tags from the iTRAQ reagents 4plex kit (AB Sciex, Foster City, CA). The samples were pooled and labeled peptides were first fractionated on an Agilent 3100 OFFGEL fractionator (GE Healthcare, Chalfont St. Giles, UK) and then analyzed on a LTQ Orbitrap Velos mass spectrometer (Thermo Electron, San Jose, CA). Peak lists from MS analysis were searched against Uniprot_Swissprot database (version 2011_2) using Phenyx protein identification software (GeneBio, Geneva, Switzerland). For protein quantiﬁcation, iTRAQ reporter peak intensities were normalized using values obtained for bovine beta-lactoglobulin (LACB) spiked into samples. Inter-experimental ratios were calculated and the cut-off chosen for defining a protein as overexpressed was fixed at 1.5.

*WS-1 RT-qPCR analysis after Cisplatin-treatment*

WS-1 cell pellets were washed once with PBS and lysed with 350 µl RLT buffer. RNA was extracted from cells using an RNeasy 96 Kit (Qiagen, 74181) according to the manufacturer's instructions (RNeasy 96 protocol for Isolation of total RNA from animal cells, using spin technology). cDNA was synthesized with Transcriptor First Strand cDNA Synthesis Kit from Roche (04 897 030 001). RNA was mixed with oligo(dT)_18_ Primer 2.5µM and random hexamer primer 60µM, and denatured by heating to 65°C for 10 min. Afterwards, 1x reaction buffer (8mM MgCl2), 20U RNase Inhibitor, 1mM dNTP and 10U Transcriptor reverse transcriptase were added and the synthesis reaction was performed for 10min at 25°C, followed by 50°C for 60 min and stopped by heating at 85°C for 5min.

QPCR reactions were performed for relative cDNA quantification for genes of interest using LC480 Probes Master (Roche Applied Science) and specific TaqMan Probes and Primers (Applied Biosystems), according to the manufacturer's instructions. Analysis was conducted using the Δ-Δcycle threshold (ct) method, which determined fold changes in gene expression relative to a control-treated sample. Each analysis reaction was performed in duplicate, with 3 samples per condition. Gene expression was normalized to glyceraldehyde-3-phosphate dehydrogenase (GAPDH), which was used as an internal reference gene and was measured with the LightCycler 480 Instrument (Roche Applied Science).

Catalogue numbers for primers and probes:

Alpha smooth muscle actin 2 ACTA2 Hs00426835_g1

connective tissue growth factor CTGF Hs00170014_m1

inhibitor of DNA binding 1 ID1 Hs03676575_s1

collagen, type I, alpha 1 Col1A1 Hs00164004_m1

transforming growth factor, beta 1 TGFB1 Hs00998133_m1

glyceraldehyde-3-phosphate dehydrogenase GAPDH Hs99999905_m1

***References***

1. Subramanian A, Tamayo P, Mootha VK, Mukherjee S, Ebert BL, et al. (2005) Gene set enrichment analysis: a knowledge-based approach for interpreting genome-wide expression profiles. Proc Natl Acad Sci U S A 102: 15545-15550.

2. Lenburg ME, Liou LS, Gerry NP, Frampton GM, Cohen HT, et al. (2003) Previously unidentified changes in renal cell carcinoma gene expression identified by parametric analysis of microarray data. BMC cancer 3: 31.

3. Fetaud V, Frossard JL, Farina A, Pastor CM, Buhler L, et al. (2008) Proteomic profiling in an animal model of acute pancreatitis. Proteomics 8: 3621-3631.
