# Supplementary material for: Epithelial Cells as Active Player In Fibrosis: Findings from an In Vitro Model
Source: PLoS One. 2013 Feb 14;8(2):e56575. doi: 10.1371/journal.pone.0056575 (PMC3572957; doi:10.1371/journal.pone.0056575)
Supplement: Table S1 — Proteomic analysis of Cisplatin-injured epithelial cells at different time-points. Asterisk indicates upregulated genes present also in figure 1G, - indicates Unidentified protein, NQ indicates Identified by not quantified proteins and NS indicates Not statistically significantly differentially modulated protein. Differential expression measured within each time-point is indicated with the following color code. (DOC) [file pone.0056575.s004.doc]

|  |  |  |  |  |  | |  |  |  |  | |  |  |  | |  |
| --- | --- | --- | --- | --- | --- | --- | --- | --- | --- | --- | --- | --- | --- | --- | --- | --- |
|  |  |  |  |  | **24h** | | |  | **48h** | | |  | **72h** | | |  |
|  | **Gene name** |  | **Protein ID** |  | **Cis Low vs Ctrl** | **Cis High vs Ctrl** | |  | **Cis Low vs Ctrl** | | **Cis High vs Ctrl** |  | **Cis Low vs Ctrl** | | **Cis High vs Ctrl** |  |
|  | ISG15 | ***** | ISG15_HUMAN |  | NS | NS | |  | **2.4** | | **3.3** |  | **2.8** | | **4.8** |  |
|  | OASL | ***** | OASL_HUMAN |  | NQ | NQ | |  | **2.2** | | **2.5** |  | **2.7** | | **4.2** |  |
|  | IFIT2 | ***** | IFIT2_HUMAN |  | - | - | |  | **3.1** | | **3.6** |  | **2.8** | | **3.9** |  |
|  | IFIT1 | ***** | IFIT1_HUMAN |  | - | - | |  | **2.0** | | **2.2** |  | **2.8** | | **3.3** |  |
|  | TP53I3 | ***** | QORX_HUMAN |  | - | - | |  | NQ | | NQ |  | **2.1** | | **3.1** |  |
|  | IFIT3 | ***** | IFIT3_HUMAN |  | - | - | |  | **2.2** | | **2.5** |  | **2.1** | | **3.1** |  |
|  | TOP2A |  | TOP2A_HUMAN |  | NQ | NQ | |  | **2.2** | | **2.5** |  | **1.7** | | **2.6** |  |
|  | RRM2B |  | RIR2B_HUMAN |  | - | - | |  | - | | - |  | **1.7** | | **2.3** |  |
|  | LIMA1 |  | LIMA1_HUMAN |  | NS | NS | |  | **1.2** | | **1.6** |  | **1.3** | | **2.3** |  |
|  | DDX60 |  | DDX60_HUMAN |  | - | - | |  | NQ | | NQ |  | **2.3** | | **2.3** |  |
|  | DDX58 |  | DDX58_HUMAN |  | - | - | |  | **1.7** | | **1.8** |  | **2.0** | | **2.2** |  |
|  | FDXR |  | ADRO_HUMAN |  | - | - | |  | - | | - |  | **1.9** | | **2.1** |  |
|  | SUPT16H |  | SP16H_HUMAN |  | **1.3** | **1.3** | |  | **1.3** | | **1.5** |  | NS | | **1.9** |  |
|  | MX1 | ***** | MX1_HUMAN |  | NQ | NQ | |  | NQ | | NQ |  | **2.0** | | **1.9** |  |
|  | FHL2 |  | FHL2_HUMAN |  | NS | NS | |  | **1.2** | | **1.4** |  | **1.3** | | **1.9** |  |
|  | ZNFX1 |  | ZNFX1_HUMAN |  | - | - | |  | **1.3** | | NS |  | **1.5** | | **1.7** |  |
|  | HPD |  | HPPD_HUMAN |  | - | - | |  | NS | | NS |  | **1.8** | | **1.6** |  |
|  | HSPA12A |  | HS12A_HUMAN |  | NQ | NQ | |  | - | | - |  | **1.4** | | **1.6** |  |
|  | HSPA12B |  | HS12B_HUMAN |  | NQ | NQ | |  | - | | - |  | **1.4** | | **1.6** |  |
|  | TUBB3 |  | TBB3_HUMAN |  | **1.1** | **1.1** | |  | **1.1** | | **1.2** |  | **1.3** | | **1.6** |  |
|  | HN1 |  | HN1_HUMAN |  | NS | NS | |  | NQ | | NQ |  | **1.3** | | **1.6** |  |
|  | SDPR |  | SDPR_HUMAN |  | **0.9** | **0.9** | |  | **1.2** | | **1.2** |  | **1.4** | | **1.5** |  |
|  | IRF2BP2 |  | I2BP2_HUMAN |  | - | - | |  | NQ | | NQ |  | **1.6** | | **1.5** |  |
|  | C2orf71 |  | CB071_HUMAN |  | **1.3** | **1.5** | |  | NQ | | NQ |  | NS | | **1.5** |  |
|  | ZC3HAV1 |  | ZCCHV_HUMAN |  | NS | NS | |  | **1.2** | | NS |  | **1.3** | | **1.5** |  |
|  | TPD52 |  | TPD52_HUMAN |  | **1.1** | **1.1** | |  | NS | | NS |  | **1.8** | | **1.5** |  |
|  | CCNB1 |  | CCNB1_HUMAN |  | **1.5** | **1.5** | |  | **1.6** | | **1.8** |  | **1.4** | | **1.4** |  |
|  | ALB |  | ALBU_HUMAN |  | **1.1** | **1.4** | |  | **1.4** | | **1.8** |  | **0.9** | | **1.4** |  |
|  | TK1 |  | KITH_HUMAN |  | **1.6** | **1.6** | |  | **1.3** | | **1.7** |  | **1.2** | | **1.3** |  |
|  | NOLC1 |  | NOLC1_HUMAN |  | **1.1** | **0.9** | |  | NS | | **0.8** |  | **2.1** | | **1.3** |  |
|  | H3F3A |  | H33_HUMAN |  | **1.0** | **1.3** | |  | **1.4** | | **1.5** |  | NS | | **1.3** |  |
|  | HIST1H2AA |  | H2A1A_HUMAN |  | - | - | |  | **1.3** | | **1.5** |  | **0.8** | | **1.3** |  |
|  | HK1 |  | HXK1_HUMAN |  | **1.2** | NS | |  | **1.1** | | **1.1** |  | **1.5** | | **1.2** |  |
|  | RACGAP1 |  | RGAP1_HUMAN |  | **1.5** | **1.7** | |  | - | | - |  | **1.1** | | **1.2** |  |
|  | KRT8 |  | K2C8_HUMAN |  | **0.6** | **0.9** | |  | **1.3** | | **1.6** |  | **0.6** | | **1.1** |  |
|  | KRT18 |  | K1C18_HUMAN |  | **0.6** | **0.9** | |  | **1.2** | | **1.5** |  | **0.6** | | **1.1** |  |
|  | RRM2 |  | RIR2_HUMAN |  | **1.3** | **1.7** | |  | NS | | **1.1** |  | NS | | **0.9** |  |
|  | VDAC2 |  | VDAC2_HUMAN |  | NQ | NQ | |  | **1.5** | | **1.4** |  | NS | | **0.9** |  |
|  | VIM |  | VIME_HUMAN |  | **0.6** | **0.8** | |  | **1.5** | | **1.6** |  | **0.6** | | **0.8** |  |
|  | RABGAP1 |  | RBGP1_HUMAN |  | **1.2** | **1.6** | |  | **1.2** | | NS |  | NS | | **0.8** |  |
|  | MKI67 |  | KI67_HUMAN |  | NQ | NQ | |  | NQ | | NQ |  | **1.7** | | NS |  |
|  | APAF1 |  | APAF_HUMAN |  | NQ | NQ | |  | - | | - |  | **1.1** | | NS |  |
|  | EPPK1 |  | EPIPL_HUMAN |  | - | - | |  | NQ | | NQ |  | **1.1** | | NS |  |
|  | ARFGEF2 |  | BIG2_HUMAN |  | **1.4** | **1.5** | |  | NQ | | NQ |  | NQ | | NQ |  |
|  | HMMR |  | HMMR_HUMAN |  | NQ | NQ | |  | **1.9** | | **2.0** |  | NQ | | NQ |  |
|  | AURKA |  | STK6_HUMAN |  | **1.5** | **1.4** | |  | **1.8** | | **2.0** |  | NQ | | NQ |  |
|  | FAM83D |  | FA83D_HUMAN |  | - | - | |  | **1.6** | | **1.7** |  | NQ | | NQ |  |
|  | DBN1 |  | DREB_HUMAN |  | 1.0 | NS | |  | **1.1** | | **1.5** |  | - | | - |  |
|  |  |  |  |  |  |  | |  |  | |  |  |  | |  |  |

**Table S1: Proteomic analysis of Cisplatin-injured epithelial cells at different time-points**

|  |  |  |  |  |
| --- | --- | --- | --- | --- |
| **≤ 1.5** | **≤ 2.5** | **≤ 3.5** | **≤ 4.5** | **≤ 5.5** |
